# Supplementary material for: Long ssRNA undergoes continuous compaction in the presence of polyvalent cations
Source: Biophys J. 2023 Jul 27;122(17):3469–75. doi: 10.1016/j.bpj.2023.07.022 (PMC10502455; doi:10.1016/j.bpj.2023.07.022)
Supplement: Document S1. Figures S1–S4 [file mmc1.pdf]

Biophysical Journal, Volume *122*

Supplemental information

Long ssRNA undergoes continuous compaction in the presence of polyvalent cations

Ana Luisa Duran-Meza, Liya Oster, Richard Sportsman, Martin Phillips, Charles M. Knobler, and William M. Gelbart

SUPPORTING MATERIAL (SI)

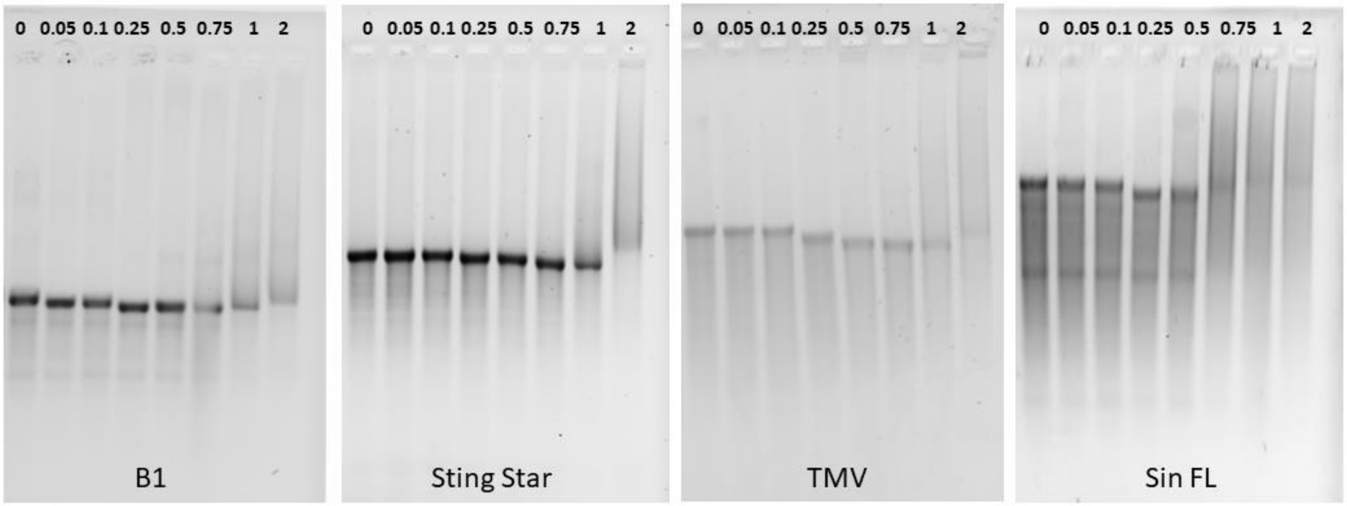

FIGURE S1 Eight-lane 0.8% agarose gels in TAE buffer, for (from left to right) B1 RNA (3234 nt), “Star” RNA (4628 nt), TMV RNA (6395 nt), and Sin FL RNA (11703 nt), for successively higher spermine:RNA charge ratios. Each gel contains 8 lanes labeled 0, 0.05, 0.1, 0.25, 0.5, 0.75, 1, and 2 for the spermine:RNA charge ratio. Note that in each case the molecules move faster with increasing charge ratio, because of their smaller size, until they begin to get stuck in the well at high enough charge ratio because of intermolecular aggregation.

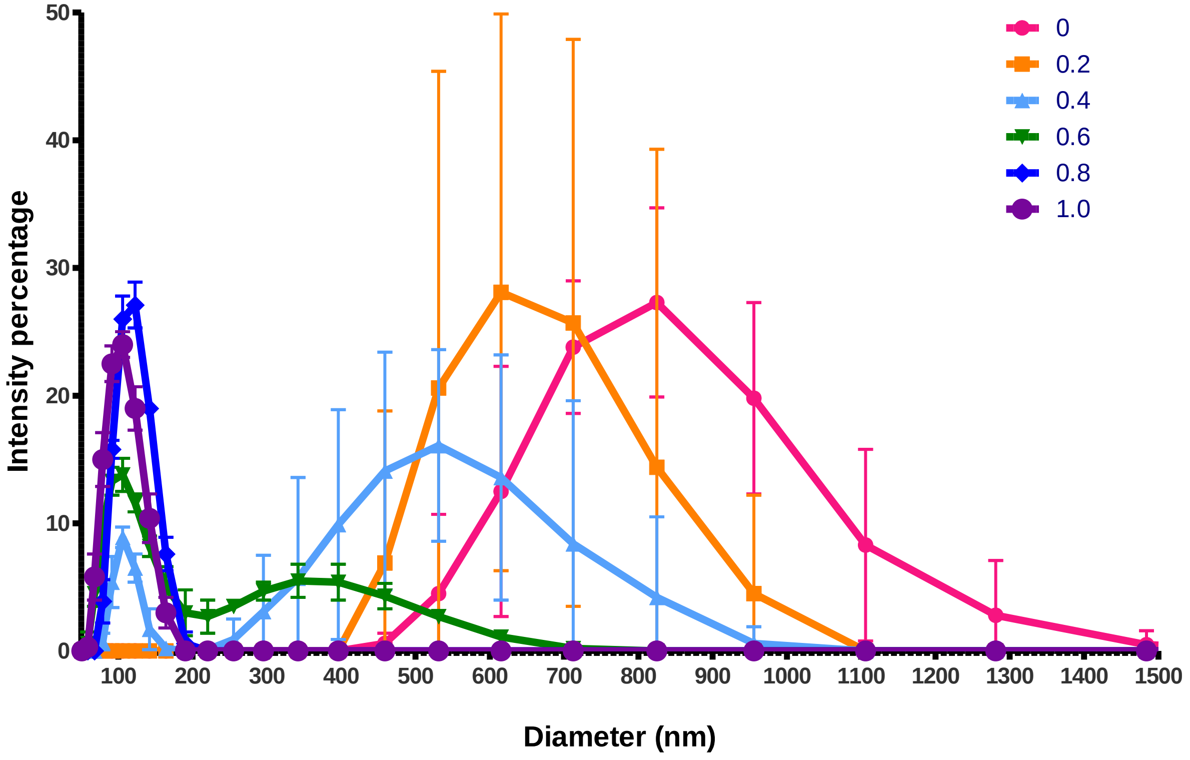

FIGURE S2 Size distributions measured by dynamic light scattering for Lambda DNA, for different spermine:DNA charge ratios, showing two-phase coexistence (bimodal distributions) for intermediate values (0.4:1 and 0.6:1), and unimodal distributions for higher (0.8:1 and 1:1) and lower (0:1 and 0.2:1) values.

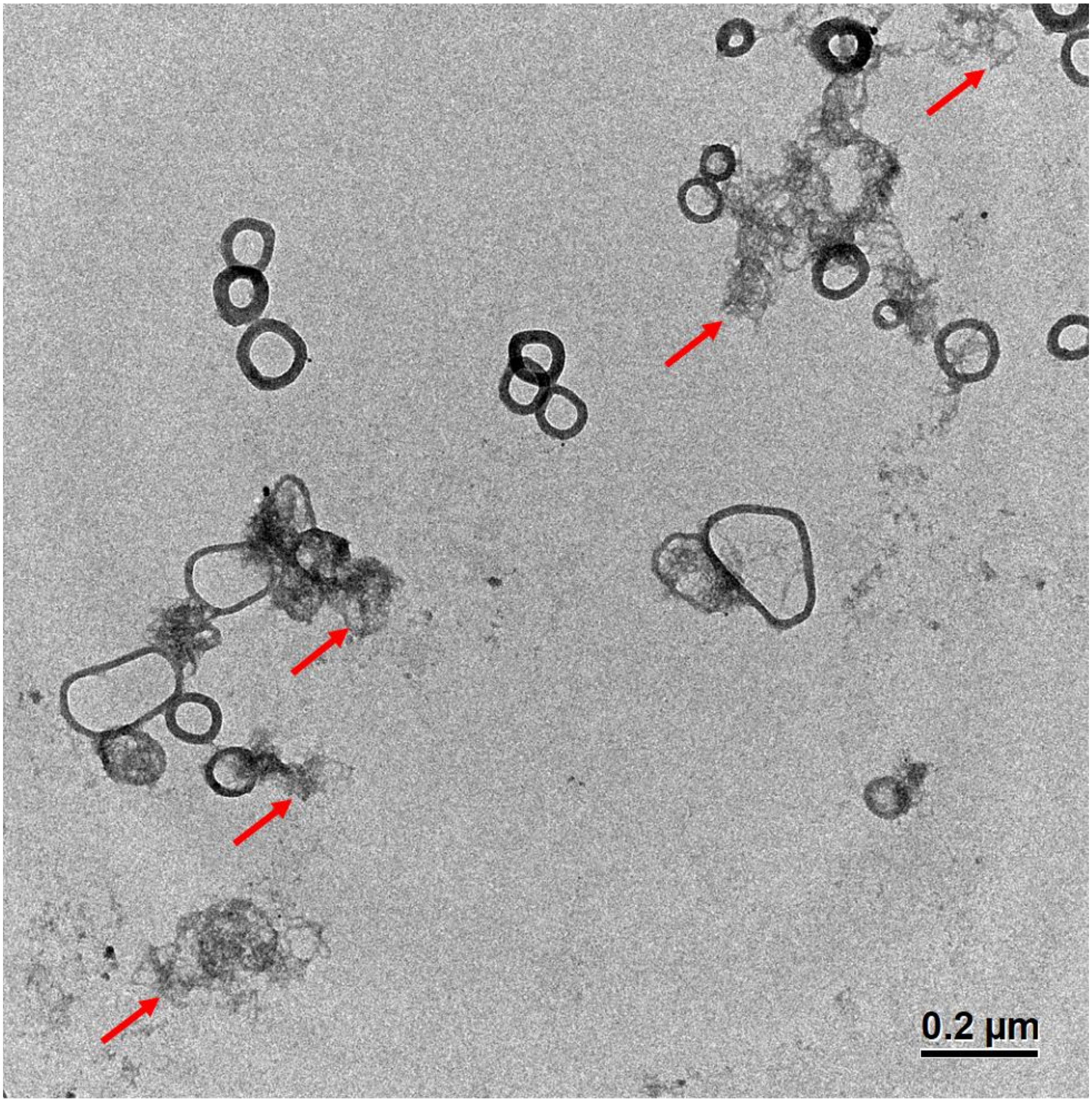

FIGURE S3 Electron micrograph of (48.5 kpb) Lambda DNA with a spermine:DNA charge ratio of 0.6:1, showing several instances, in the same field of view, of coexistence of uncondensed disordered DNA (see arrow) with strongly-condensed/toroidally-close-packed molecule**.** An aliquot of 6 μL of of DNA with spermine in TE buffer at a concentration of 0.2 mg/mL was deposited on glow-discharged carbon-coated copper (200-mesh) PELCO Pinpointer grids (Ted Pella, USA). After 1 m, the grids were blotted with Whatman filter paper, and then stained with 6 μL of 2 % uranyl acetate for 1 min followed by complete stain removal and storage in a desiccator overnight. Micrographs were acquired using a Tecnai G2 TF20 High-Resolution electron microscope (FEI, USA) with an accelerating voltage of 200 kV. Images were collected with a TIETZ F415MP 16-megapixel CCD camera (4000 by 4000 pixels, pixel size 15 μm).

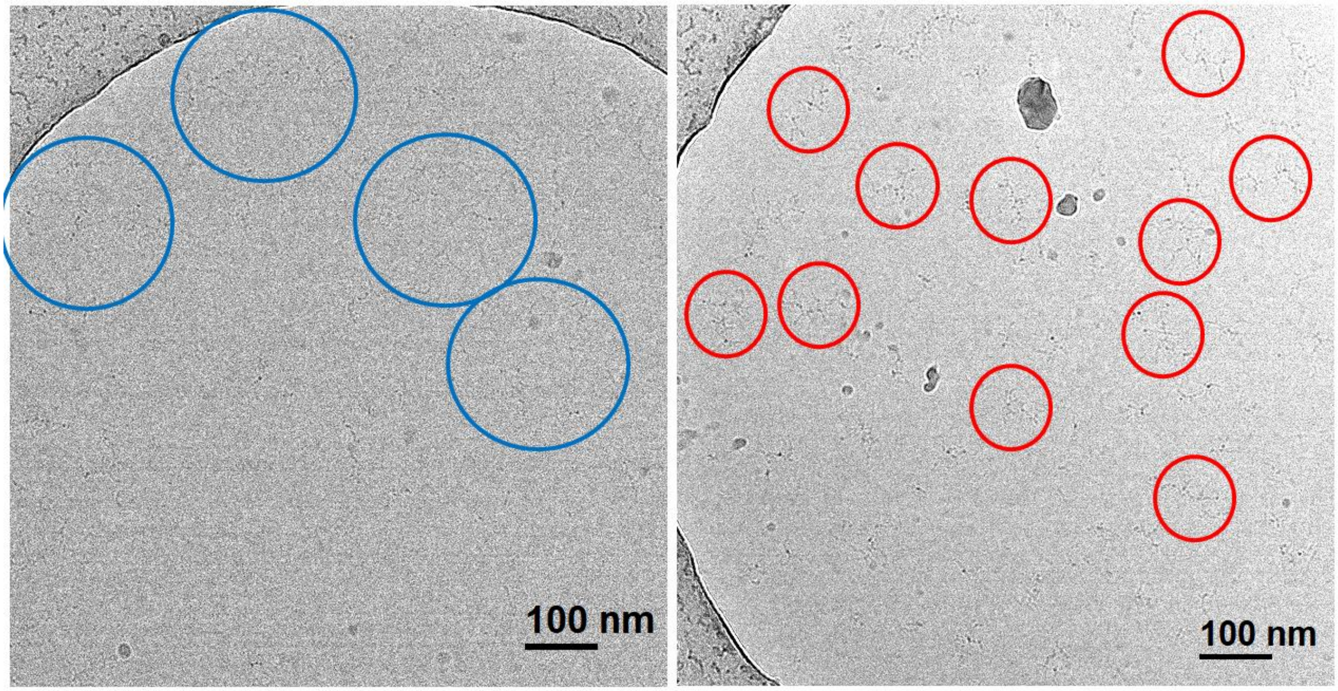

FIGURE S4 Electron micrographs of Sin FL ssRNA in the absence (left) and presence (right) of spermine (at a charge ratio of 1). Blue and red circles are guides-to-the-eye highlighting the differences in size of the molecule under these conditions of polyvalent cation. The molecules are barely visible because of their low density: no discontinuous condensation has occurred – rather, the only effect of the spermine is to progressively and weakly compactify the RNA. An aliquot of 3 μL of RNA with spermine in water at a concentration of 1 mg/mL was applied to a QUNTIFOIL holey carbon grid (2/1, 300 mesh), which was glow-discharged for 60 s with a PELCO easiGlow system.. The grid was blotted and plunged-frozen from 4°C under 100% humidity into liquid ethane with a Vitrobot IV. The grid was stored in liquid nitrogen until imaging. Cryo-Micrographs were acquired using a Tecnai G2 TF20 High-Resolution electron microscope (FEI, USA) with an accelerating voltage of 200 kV. Images were collected with a TIETZ F415MP 16-megapixel CCD camera (4000 by 4000 pixels, pixel size 15 μm).
