# Supplementary material for: Long ssRNA undergoes continuous compaction in the presence of polyvalent cations
Source: Biophys J. 2023 Jul 27;122(17):3469–75. doi: 10.1016/j.bpj.2023.07.022 (PMC10502455; doi:10.1016/j.bpj.2023.07.022)
Supplement: Document S2. Article plus supporting material [file mmc2.pdf]

Biophysical Journal
Article

Biophysical Society

Long ssRNA undergoes continuous compaction in the presence of polyvalent cations

Ana Luisa Duran-Meza,[1] Liya Oster,[1] Richard Sportsman,[1] Martin Phillips,[1] Charles M. Knobler,[1,*] and William M. Gelbart[1,2,3]
[1]Department of Chemistry and Biochemistry, UCLA, Los Angeles, California; [2]Molecular Biology Institute, UCLA, Los Angeles, California; and [3]California NanoSystems Institute, UCLA, Los Angeles, California

ABSTRACT In the presence of polyvalent cations, long double-stranded DNA (dsDNA) in dilute solution undergoes a single-molecule, first-order, phase transition ("condensation"), a phenomenon that has been documented and analyzed by many years of experimental and theoretical studies. There has been no systematic effort, however, to determine whether long single-stranded RNA (ssRNA) shows an analogous behavior. In this study, using dynamic light scattering, analytical ultracentrifugation, and gel electrophoresis, we examine the effects of increasing polyvalent cation concentrations on the effective size of long ssRNAs ranging from 3000 to 12,000 nucleotides. Our results indicate that ssRNA does not undergo a discontinuous condensation as does dsDNA but rather a "continuous" decrease in size with increasing polyvalent cation concentration. And, instead of the 10-fold decrease in size shown by long dsDNA, we document a 50% decrease, as demonstrated for a range of lengths and sequences of ssRNA.

SIGNIFICANCE The 3D sizes of long (thousands of nucleotides long) single-stranded RNA are important in many different biological contexts, ranging from confined RNA genomes in viral capsids to nuclear export of mRNAs, with size being determined partly by large-scale sequence-specific secondary/tertiary structure and partly by nonspecific electrostatic interactions mediated by mono-, di-, and polyvalent counterions. We report here the first, to the best of our knowledge, systematic study of the effects of polyvalent cations on the compaction of long single-stranded RNA molecules and show that it is continuous, in contrast to the well-studied case of double-stranded DNA, which undergoes a discontinuous phase transition—condensation—as a function of increasing tri- and tetravalent counterion concentration.

INTRODUCTION

Double-stranded (ds) DNAs with contour lengths long compared with their persistence length behave under physiological solution conditions like disordered self-repelling polymers. In the presence of sufficiently high concentrations of polyvalent cations, however, they condense discontinuously into circumferentially wound, self-attracting, toroidal structures that are hexagonally close packed along most of their length. This phenomenon, involving the appearance of an effective attraction of the DNA for itself, has been addressed experimentally in many papers (see, for example, (1–5)) and has been accounted for by the breakdown of mean-field (Poisson-Boltzmann and Debye-Hückel) theories of counterion effects and by the role of correlations between polyvalent cations (see, for example, (6–12)). In the case of long single-stranded (ss) RNA, on the other hand, the only studies of the effects of polyvalent cations have involved isolated measurements of RNA size in the presence of one or another di-, tri-, or tetravalent cation (see, for example, (13–15)), with no attempt to characterize the "general" nature (e.g., continuous versus discontinuous) of the dependence of size on the concentration of polyvalent cation.

A great deal of work has been devoted to elucidation of the effects of counterions—especially divalent cations—on the evolution of secondary and tertiary structure of short RNA molecules that fold into specific structural and

Submitted May 2, 2023, and accepted for publication July 25, 2023.

*Correspondence: knobler@chem.ucla.edu

Liya Oster's present address is Biophysics Graduate Program, Department of Bioengineering, UC Berkeley

Richard Sportsman's present address is Sportsman Consulting, LLC, San Diego, California

Editor: Mark Williams.

https://doi.org/10.1016/j.bpj.2023.07.022

© 2023 Biophysical Society.

This is an open access article under the CC BY license (http://creativecommons.org/licenses/by/4.0/).

enzymatic motifs (16–19). Moghaddam et al., for example, using small-angle x-ray scattering, have demonstrated different pathways for the multivalent-cation-induced compactions of two different ribozymes (20). In the case of RNAs that are ~3000 nt or longer, however, such as the single-stranded genomes of most viruses, the RNA molecules are not associated with any one secondary/tertiary structure but rather with a large "ensemble" of them, all of which are thermally accessible (21–26). Here the relevant question becomes: how does the ensemble-averaged size depend on the overall length and sequence of the RNA and on the concentration of polyvalent cation? In the present work, we report several sets of measurements—specifically, dynamic light scattering, analytical ultracentrifugation, and gel electrophoresis—that allow us to conclude that the size of a long ssRNA molecule in dilute solution decreases "continuously," and by no more than 50%, as the concentration of polyvalent cation (spermine here) is increased. This result is in qualitative contrast to the situation with long dsDNA, where—as we show by documenting the presence of two distinct coexisting states of the DNA over a range of spermine concentrations—the decrease in size is as large as 10-fold and is discontinuous. The continuous compaction of ssRNA is confirmed for different nucleotide sequences and lengths (from 3000 to 12,000 nt) and for cationic/anionic charge ratios varying from 0 to 3.

MATERIALS AND METHODS

Reagents

Five different ssRNA molecules were studied, ranging in length from 3234 to 11,703 nt: "B1" (brome mosaic virus (BMV) RNA1), 3234 nt, is the longest of the three molecules making up the genome of the BMV virus (27); "Sting Star (Star)," 4628 nt, is a genetic fusion of RNA1 of the Nodamura virus (28) and the gene encoding the constitutively active form of the STING protein (29); "B1B3," 5317 nt, is a genetic fusion of BMV RNA1 and BMV RNA3, with the 3′ end of RNA1 fused to the 5′ end of RNA3 (30); "TMV," 6395 nt, is the genome of the tobacco mosaic virus (TMV) (31); and Sin FL is the full-length (FL) genome of the Sindbis virus (32). These RNAs were obtained by transcription, respectively, of the linear plasmids pT7B1 (B1), Nod1-STING* (Star), pRDCT7B1B3 (B1B3), p30B (TMV), and pTE12 (Sin FL), using a MEGAscript T7 or SP6 Transcription Kit (Thermo Fisher, USA), eluted in water, and frozen at –80°C until use. The ssRNA lengths were corroborated by running a 0.8% agarose gel in TAE buffer (40 mM Tris-HCl, pH 8, 20 mM acetic acid, 1 mM EDTA).

The appearance of a second band in the transcription mix of B1B3 necessitated additional purification. It was loaded into the central wells of 0.8% agarose gel, flanked on either side by aliquots that were stained with ethidium bromide. Using the labeled RNA bands as a ruler, the unstained RNA was excised from the gel and extracted from the excised slice by electroelution into a dialysis bag. The extracted RNA was then washed five times in TE buffer (10 mM Tris-HCl, pH 7.5, 1 mM EDTA) using a 100-kDa Amicon Ultra-0.5 centrifugal filter spun at 7000 *g* at 4°C for 10 min, with each spin involving a ~10-fold change in volume, resulting in a buffer/solute exchange of ~100,000-fold.

Spermine was obtained from Sigma (Sigma, USA). To ensure the reproducibility of the measurements, aliquots were prepared by dissolving spermine in degassed water at a concentration of 0.1 mg/mL, which were then purged with argon and stored at −80°C for no longer than a month.

Dynamic light scattering

Dynamic light scattering (DLS) measurements were performed with a Malvern Zetasizer Nano ZN (Malvern Panalytical, UK) at 25°C using 40 μL cuvettes. RNA molecules were "monomerized" by heating them to 90°C at a rate of 1°C/s, holding the sample at 90°C for 1 s, and then slowly cooling at a rate of 1°C/s. 500 ng of RNA was incubated in 10 mM Tris-HCl buffer (pH 7.2, 10 mM), which had been degassed by bath sonication and argon purging with different concentrations of spermine for 30 min at 4°C in a total volume of 50 μL (corresponding to an RNA concentration of 0.01 mg/mL). Each data point is the mean of 100 measurements.

Analytical ultracentrifugation

Changes in sedimentation coefficient of B1B3 with spermine were measured using an Optima XL-A analytical ultracentrifuge (AUC) (Beckman-Coulter, USA). The concentration of the RNA in these experiments was 0.008 μg/mL RNA, with a total of 400 μL of solution, to maximize signal and to minimize the chance of RNA and spermine forming multi-RNA aggregates. The buffer was 10 mM Tris-HCl (pH 7.5). The centrifugation speed and time were adjusted in each run to account for the change in sedimentation velocity of each RNA and spermine sample.

Gel electrophoresis

Gel electrophoresis experiments were performed in an electrophoresis tank Owl EasyCast B1A Mini Gel Electrophoresis Systems (Thermo Fisher, USA), using 0.8% agarose in TAE (40 mM Tris-HCl, pH 8, 20 mM acetic acid, 1 mM EDTA). The control consisted of RNA fully denatured by heating to 65°C for 10 min and then mixed with 2X RNA Gel Loading dye and cooled to 4°C. Samples containing spermine were thermally denatured (90°C for 1 s, then slowly cooled at a rate of 1 °C/s) and incubated with spermine at each of several spermine/RNA charge ratios. More explicitly, 1.5 μg RNA was mixed with spermine and incubated at 4°C for 30 min, with cationic/anionic charge ratios of 0, 0.01, 0.025, 0.05, 0.075, 0.10, 0.25, 0.50, 0.75, 1.0, and 2.0. The final sample volumes were 20 μL. They were prestained with 2 μL 200X GelRed, and the gel electrophoresis was performed at 100 V for 1.5 h.

RESULTS

Hydrodynamic diameters of ssRNA, ranging in length from 3000 to 12,000 nt, decrease "continuously" upon increase of spermine concentration

Fig. 1 *A* shows intensity-weighted size distributions measured by DLS for B1 RNA (3234 nt), for each of six different spermine/RNA charge ratios, increasing from 0 to 1.5. The 15–20 nm width of each size distribution does not arise from polydispersity in the length of the RNA molecules but rather from the polydispersity (ensemble) of secondary/tertiary structures associated with any long ssRNA molecule (21–26). The presence of only a single peak in each of the distributions reflects the fact that for each spermine/RNA charge ratio over this broad

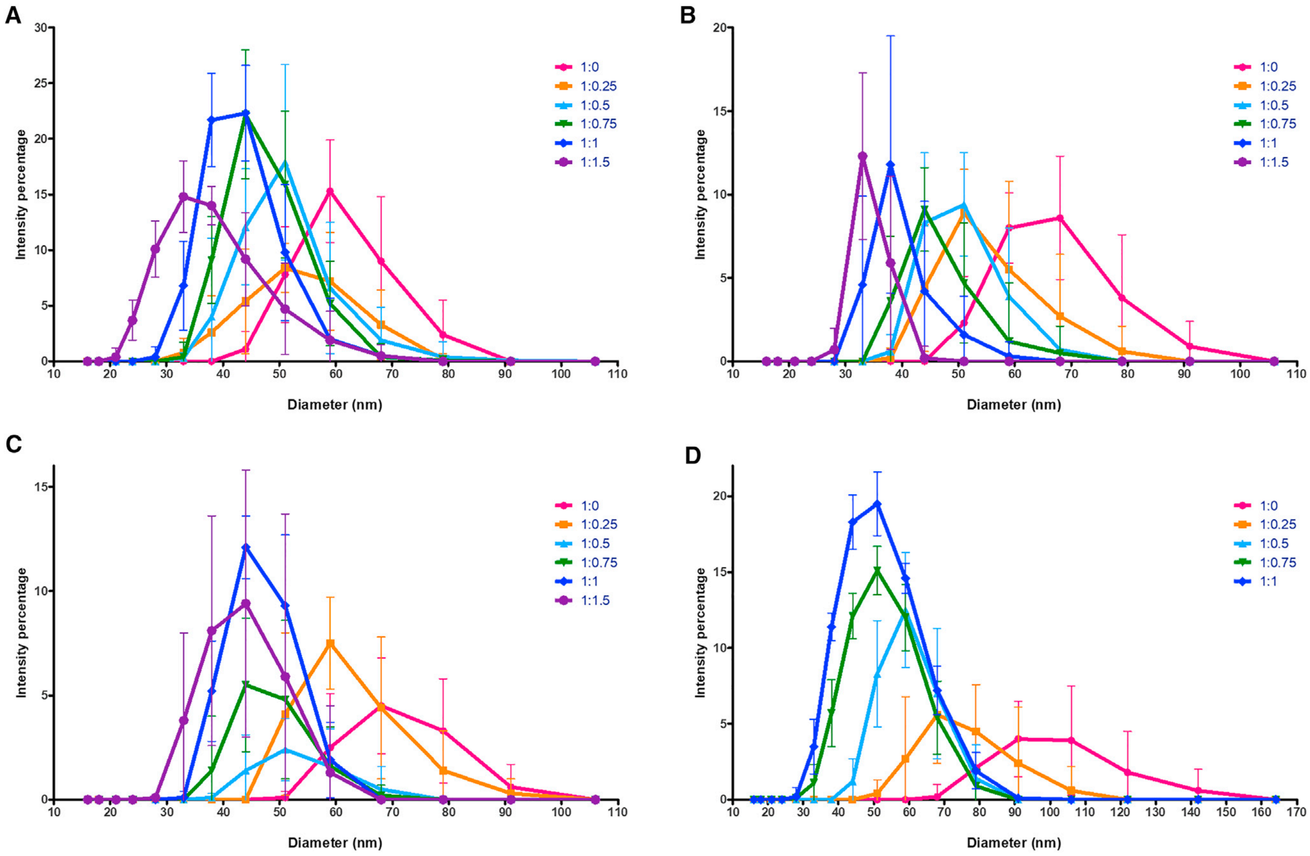

FIGURE 1 DLS-measured size distributions for each of several increasing spermine/RNA charge ratios, ranging from 0 to 1.5, for B1 RNA (3234 nt) (see 1*A*), Sting Star RNA (4268 nt) (1*B*), and TMV RNA (6395 nt) (1*C*), and from 0 to 1 for Sin FL RNA (11703nt) (1*D*). Each data point is the average of 100 measurements, and ten data points were taken for each RNA at each charge ratio and are plotted here as their triplicate average, with bars representing standard deviations. To see this figure in color, go online.

range there do not arise two different, coexisting, ensembles of RNA conformations. Exactly the same behavior is seen for three additional lengths and sequences of ssRNA (see Fig. 1 *B–D*), and in each case, the widths of the size distributions are seen to decrease with increasing spermine/RNA charge ratio. The fact that the size distributions remain unimodal for all charge ratios, for each of the several different molecules, and that the most probable/average size decreases continuously with increasing charge ratio, reflects the absence of a first-order condensation transition. This is in qualitative contrast to the situation for long dsDNA, where (see below) distinctly "bimodal" size distributions are observed for intermediate values of the cationic/anionic charge ratio, corresponding to a two-state coexistence involving uncondensed and compacted dsDNA.

DLS measurements of RNA size distributions like those reported in Fig. 1 for spermine/RNA (cationic/anionic) charge ratios of 0, 0.25, 0.5, 0.75, 1.0, and 1.5 were also performed at the higher values of 2.0, 2.5, and 3.0. Fig. 2 shows a plot of the corresponding mean sizes (the average hydrodynamic diameters) as a function of charge ratio over the extended range 0–3, for the B1, Star, TMV, and Sin FL RNA molecules. From these DLS measurements, we see a continuous compaction of the ssRNA, up to about a 2:1 ratio, after which the curve begins to level off. At still larger values of the charge ratio, the average size appears to begin to increase, and this is because of the onset of contributions from aggregates of the RNA molecules. The onset of intermolecular interactions—and, specifically, aggregation of RNA—at higher spermine concentrations is also seen in gel electrophoresis analyses of these RNA molecules (Fig. S1). More explicitly, for spermine/RNA charge ratios smaller than 1.5, the RNA runs as a unique distinct band in an agarose gel, whereas for higher values of the charge ratio, a significant fraction remains in the well. This is because the electrophoretic gel mobility, while inversely proportional to RNA size, is dependent on—directly proportional to—the effective "charge" on the molecules, which decreases with spermine concentration. Accordingly, these two effects of the spermine counter one another so that there is little change in electrophoretic mobility with increasing spermine (see Fig. S1), until aggregation sets in and most of the RNA remains in the well. Note from Fig. 2 that the sizes of all four molecules (except for the

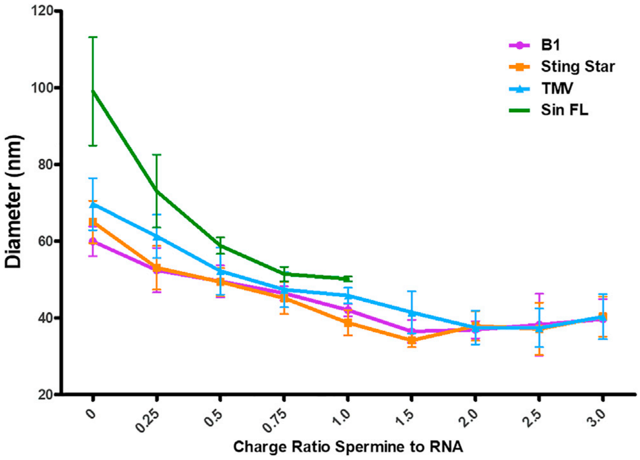

FIGURE 2 DLS-determined hydrodynamic diameters versus the spermine/RNA charge ratio, for B1 (~3200 nt), Star (~4600 nt), TMV (~6400 nt), and Sin FL (~11,700 nt) RNA compacted in TE buffer at 25°C. The RNA concentrations are 10 nM. Each data point is the average of 100 measurements, and three data points were taken for each RNA at each charge ratio and are plotted here as their triplicate average, with bars representing standard deviations. To see this figure in color, go online.

longest, Sin FL RNA) appear to level off to essentially the same values at ratios larger than 1.5, corresponding to hydrodynamic diameters of 35–40 nm; i.e., we are not able to resolve the differences in their size expected by the $N^{1/3}$ behavior predicted for these compact molecules (21–23,33).

The continuous decrease of hydrodynamic size upon increase in polyvalent cation concentration is also observed in analytical ultracentrifugation (AUC) experiments where one measures sedimentation velocities normalized by the centrifugal acceleration. Because the sedimentation velocity S is inversely proportional to the hydrodynamic diameter, we expect the compaction of RNA by spermine to appear as an increase in S. This is seen in Fig. 3, where sedimentation velocities for B1B3 RNA are plotted as a function of the spermine/RNA charge ratio and where we have inverted the vertical axis so that a continuous increase in its value can be most easily compared with the continuous decrease in molecular size (here, again, the hydrodynamic diameter) shown in Fig. 2; the maximum sedimentation velocity value corresponds to the minimum size (maximally compacted) RNA. Unlike the curve in Fig. 2, which shows a leveling off and rise at high charge ratios, however, Fig. 3 shows a continuous decrease. This difference can be attributed to the lack of aggregation in the AUC studies, which can be carried out at a thousand times lower RNA concentration.

Hydrodynamic diameters of dsDNA decrease "discontinuously" upon increase of spermine concentration

Significantly, the above behavior of ssRNA is qualitatively in contrast with what is observed when dsDNA is incubated with successively higher concentrations of spermine, corresponding to the same range of cationic/anionic charge ratios. This is seen in Fig. 4, for the case of 48.5-kbp-long dsDNA from bacteriophage lambda.

Here it is clear that for a range of spermine concentrations corresponding to charge ratios greater than 0.3 and less than 1, there are two distinctly different size distributions that coexist with one another, consistent with the system undergoing a first-order condensation phase transition. For charge ratios of 0 (i.e., no spermine, see red curve) and of 0.3 (purple curve), for example, the dsDNA sizes are unimodally distributed with most probable hydrodynamic diameters of ~800 and 500 nm, respectively. On the other hand, for a charge ratio of 0.5, the size distribution (orange) is bimodal with "coexisting" most probable diameters of ~400 and 50 nm. Similarly, for a charge ratio of 0.7 (blue curve), there

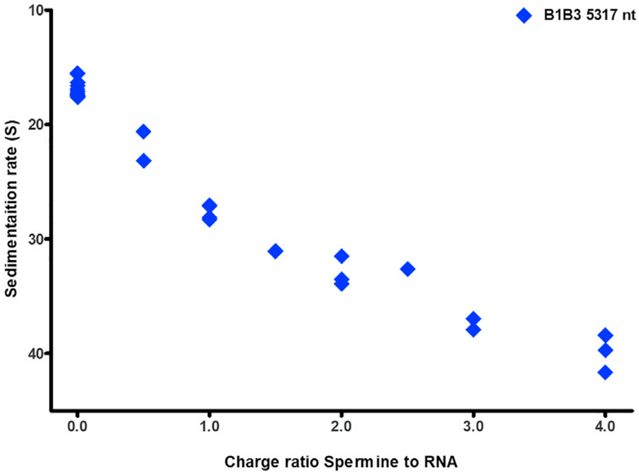

FIGURE 3 Plot of sedimentation velocities, in units of Svedbergs ($1S = 10^{-13}s$), as a function of increasing spermine/RNA charge ratio, for B1B3 RNA (~5300 nt long). To see this figure in color, go online.

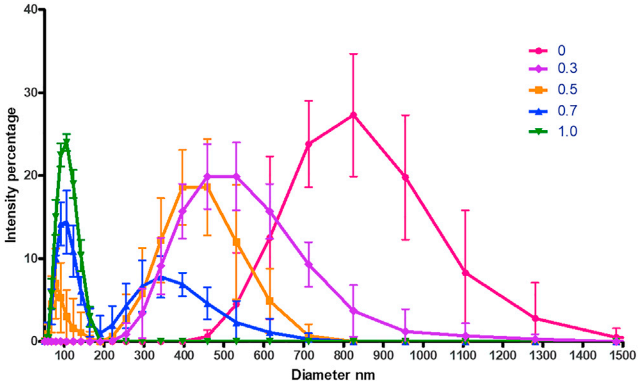

FIGURE 4 DLS-measured intensity-weighted size distributions of 48,500-bp dsDNA, at a concentration of 1 nM, for each of several spermine/DNA charge ratios, ranging from 0 to 1.0. Each data point is the average of 100 measurements, and ten data points were taken for each RNA at each charge ratio and are plotted here as their triplicate average, with bars representing standard deviations. To see this figure in color, go online.

are two sizes, corresponding to ~ 350 and 100 nm: the properties of the two states of the molecule are beginning to approach one another; indeed, for still larger spermine concentrations, the system has returned to a one-phase region (see green curve), with a single most probable size of ~100 nm. A similar progression from unimodal to bimodal to unimodal dsDNA size distributions upon increase in spermine concentration has been reported by Yoshikawa et al. for the case of 166-kbp-long dsDNA from T4 bacteriophage, as measured by fluorescence microscopy (34). Additional DLS size-distribution progressions of this kind for lambda dsDNA are included in Fig. S2. We also include several electron microscope images (see Fig. S3) of toroidally condensed DNAs each coexisting with a much larger configuration of uncondensed DNA, for a spermine/dsDNA charge ratio of 0.6. No corresponding coexistence between condensed and uncondensed states is observed for ssRNA: instead, as shown in Fig. S4, the RNA simply becomes progressively and continuously compacted as spermine concentration is increased.

DISCUSSION

We have presented data—from DLS (diffusion coefficient), analytical ultracentrifugation (sedimentation velocity), and agarose gel electrophoresis (mobility) measurements—that contrast the way in which thousands-of-nucleotides-long ssRNA and thousands-of-basepairs-long dsDNA are compacted by increasing concentrations of polyvalent cations (spermine), and we have shown that for ssRNA, the compaction is continuous, whereas for dsDNA, it is strongly discontinuous. This can be viewed in the context of the general theory of polymer collapse, according to which the compaction is continuous for linear chains and discontinuous for sufficiently stiff ones (35). Note that the persistence length of ssRNA is 1–2 nm, whereas it is as large as 50 nm for dsDNA (36). Branching, such as that associated with secondary structure formation in ssRNA, has been argued to further ensure the continuous nature of the transition (37). As a high-molecular-weight charged biomolecule, long dsDNA is basically just a stiff, linear polymer with its size scaling as square root of the mass; long ssRNA, in contrast, is flexible and effectively "branched"—because of its spontaneous self-complementary secondary structure—and hence significantly more compact, with its size scaling as the cube root of its mass (21–23,33). In the dsDNA case, the interaction between molecules or between distant parts of the same molecule is essentially that between persistence-length (~150-bp) portions of the perfect duplexes associated with their being formed from full-length complementary strands. And a great deal of work has been devoted to accounting for their self-attraction that arises in the presence of polyvalent cations and that results in a first-order condensation of the system (1–12). In the case of long ssRNA, on the other hand, about two-thirds of the nucleotides are involved in long-distance base-pairing due to self-complementarity of the molecule arising from hydrogen-bonding "attractions" between nucleotides and resulting in an effectively branched structure consisting of many single-stranded loops from which emanate two, three, or more base-paired duplex portions (21–23). Further, these duplexes are typically no longer than 5–8 bp long and accordingly are not expected to give rise to effective attractions in the presence of polyvalent cations (7,38).

Katz et al. (39) have reported that potentials of mean force between short double-stranded RNAs ~25 bp in length remain repulsive as a function of increasing concentration of polyvalent cations, in contrast to comparable-length dsDNA interactions that become attractive, attributing this qualitative difference to how cations are differentially bound in the respective major and minor grooves of the duplexes. Similarly, thermodynamic and optical measurements have shown that three 280-bp homopolymer dsDNA mixtures—polyI + polyC, polyC + polyG and polyA + polyU—are condensed discontinuously by polyamines (40). It would be interesting to look for condensation of sufficiently long (greater than many hundred basepairs) double-stranded RNA upon increase in polyvalent cation concentration, which we expect to undergo a discontinuous compaction, and to show that long single-stranded DNA is compacted continuously, as we have shown here to be the case of ssRNA. We note that Ruiz-Garcia et al. (41) have reported AFM studies of toroidal ("nanoring") condensates of ssRNA on mica surfaces in polyvalent cation aqueous solutions, but here the interactions of the RNA molecules with the negatively charged solid surface surely play a dominant role in the nature of the compaction transition. Clearly much work remains to be done to connect these related phenomena and to further elucidate the nature of the effects of polyvalent cations on different lengths of single- and double-stranded RNA and DNA.

In order to contrast the continuous compaction of long ssRNA with the well-studied discontinuous condensation of dsDNA we have confined ourselves in the present work to the role of tetravalent spermine—explicitly not including the physiologically relevant divalent magnesium. And we have chosen not only a large range of lengths of long RNAs (from ~3000 to ~12,000 nt) but also a large range of viral sequences, each with its very different evolutionary pressures relating to size and shape. "B1" RNA is an ~3200-nt gene of the multipartite plant virus BMV that is spontaneously packaged into an ~28-nm spherical capsid (23), whereas "SIN FL" is the whole, single-molecule, ~11,700-nt genome of the mammalian virus Sindbis that is spontaneously packaged into an ~40-nm spherical capsid (42). In both cases, the RNA secondary/tertiary structures ensure that the molecule will be sufficiently compact to be packaged into its spherical shell by its capsid protein, without the expenditure of any work. In addition, by design, one of the intermediate-length RNAs we have chosen to

work with is the ~6400-nt single-molecule genome of TMV, which has no evolutionary pressure on it to be compact. This is because it is spontaneously packaged into a cylindrical capsid whose length is directly proportional to its nucleotide length—and in a way that involves all secondary structure of the RNA being dismantled in the self-assembly process (43). Consistent with this fact, the TMV genome is significantly less compact than the comparable-length RNA genomes of spherical viruses (21). And yet the continuous compaction—versus discontinuous condensation—effect of spermine on the overall 3D size of the equilibrated RNAs is the same in all cases.

SUPPORTING MATERIAL

Supporting material can be found online at https://doi.org/10.1016/j.bpj.2023.07.022.

AUTHOR CONTRIBUTIONS

L.O., M.P., and R.S. planned and carried out the AUC measurements. A.L.D.-M. planned and performed the DLS and gel electrophoresis measurements. R.S. and A.L.D.-M. wrote the first drafts of sections of the paper. C.M.K. and W.M.G. directed the research and wrote the final draft of the paper.

ACKNOWLEDGMENTS

We thank Dr. Christian Beren for assistance with the cryo-electron microscopy. This research was supported by NSF Molecular and Cellular Biosciences grant #2103700. R.W.S. received support by an NIH training grant for USPHS National Research Service Award 5T32GM008496.

DECLARATION OF INTERESTS

The authors declare no competing interests.

REFERENCES

1. Bloomfield, V. A. 1997. DNA Condensation by Multivalent Cations. *Biopolymers.* 44:269–282.
2. He, S., P. G. Arscott, and V. A. Bloomfield. 2000. Condensation of DNA by Multivalent Cations: Experimental Studies of Condensation Kinetics. *Biopolymers.* 53:329–341.
3. Gelbart, W. M., R. F. Bruinsma, …, V. A. Parsegian. 2000. DNA-Inspired Electrostatics. *Phys. Today.* 53:38–44.
4. Todd, B. A., V. A. Parsegian, …, D. C. Rau. 2008. Attractive Forces between Cation Condensed DNA Double Helices. *Biophys. J.* 94:4775–4782.
5. Vilfan, I. D., C. C. Conwell, …, N. V. Hud. 2006. A Time Study of DNA Condensate Morphology: Implications Regarding the Nucleation, Growth and Equilibrium Populations of Toroids and Rods. *Biochemistry.* 45:8174–8183.
6. Oosawa, F. 1971. Polyelectrolytes. Dekker.
7. Grønbech-Jensen, N., R. J. Mashl, …, W. M. Gelbart. 1997. Counterion-induced attraction between rigid polyelectrolytes. *Phys. Rev. Lett.* 78:2477–2480.
8. Ha, B.-Y., and A. J. Liu. 1997. Counterion-mediated attraction between two like-charged rods. *Phys. Rev. Lett.* 79:1289–1292.
9. Ha, B.-Y., and A. J. Liu. 1998. Effect of Non-Pairwise-Additive Interactions on Bundles of Rodlike Polyelectrolytes. *Phys. Rev. Lett.* 81:1011–1013.
10. Levin, Y., J. J. Arenzon, and J. F. Stilck. 1999. The nature of attraction between like-charged rods. *Phys. Rev. Lett.* 83:2680.
11. Moreira, A. G., and R. R. Netz. 2000. Strong-coupling theory for counter-ion distributions. *Europhys. Lett.* 52:705–711.
12. Grosberg, A. Y., T. T. Nguyen, and B. I. Shklovskii. 2002. The Physics of Charge Inversion in Chemical and Biological Systems. *Rev. Mod. Phys.* 74:329–345.
13. Eecen, H. G., J. H. van Dierendonck, …, L. Bosch. 1985. Hydrodynamic properties of RNA–effect of multivalent cations on the sedimentation behavior of turnip yellow mosaic virus. *Biochemistry.* 24:3610–3617.
14. Borodavka, A., R. Tuma, and P. G. Stockley. 2013. A two-stage mechanism of viral RNA compaction revealed by single-molecule fluorescence. *RNA Biol.* 10:481–489.
15. Borodavka, A., S. W. Singaram, …, R. Tuma. 2016. Sizes of Long RNA Molecules Are Determined by the Branching Patterns of Their Secondary Structures. *Biophys. J.* 111:2077–2085.
16. Woodson, S. A. 2010. Compact intermediates in RNA folding. *Annu. Rev. Biophys.* 39:61–77.
17. Draper, D. E. 2004. A guide to ions and RNA structure. *RNA.* 10:335–343.
18. Nguyen, H. T., N. Hori, and D. Thirumalai. 2019. Theory and simulations for RNA folding in mixtures of monovalent and divalent cations. *Proc. Natl. Acad. Sci. USA.* 116:21022–21030.
19. Takamoto, K., R. Das, …, M. R. Chance. 2004. Principles of RNA Compaction: Insights from the Equilibrium Folding Pathway of the P4-P6 RNA Domain in Monovalent Cations. *J. Mol. Biol.* 343:1195–1206.
20. Moghaddam, S., G. Caliskan, …, S. A. Woodson. 2009. Metal Ion Dependence of Cooperative Collapse Transitions in RNA. *J. Mol. Biol.* 393:753–764.
21. Yoffe, A. M., P. Prinsen, …, A. Ben-Shaul. 2008. Predicting the sizes of large RNA molecules. *Proc. Natl. Acad. Sci. USA.* 105:16153–16158.
22. Fang, L. T., W. M. Gelbart, and A. Ben-Shaul. 2011. The size of RNA as an ideal branched polymer. *J. Chem. Phys.* 135:155105.
23. Fang, L. T., A. M. Yoffe, …, A. Ben-Shaul. 2011. A sequential folding model predicts length-independent secondary structure properties of long ssRNA. *J. Phys. Chem. B.* 115:3193–3199.
24. van der Schoot, P., and R. Zandi. 2013. Impact of the topology of RNAs on their encapsulation by virus coat proteins. *J. Biol. Phys.* 39:289–299.
25. Gopal, A., D. E. Egecioglu, …, W. M. Gelbart. 2014. Viral RNAs Are Unusually Compact. *PLoS One.* 9, e105875.
26. Garmann, R. F., A. Gopal, …, S. C. Harvey. 2015. Visualizing the Global Secondary Structure of a Viral Genome with Cryo-Electron Microscopy. *RNA.* 21:1–10.
27. Rao, A. L. N. 2006. Genome Packaging by Spherical Plant RNA Viruses. *Annu. Rev. Phytopathol.* 44:61–87.
28. Ball, L. A., and K. L. Johnson. 1999. Reverse Genetics of Nodaviruses. *Adv. Virus Res.* 53:229–244.
29. Liu, W., M. G. Alameh, …, J. You. 2022. Lipid Nanoparticles Delivering Constitutively Active STING mRNA to Stimulate Antitumor Immunity. *Int. J. Mol. Sci.* 23, 14504.
30. Cadena-Nava, R. D., M. Comas-Garcia, …, W. M. Gelbart. 2012. Self-assembly of viral capsid protein and RNA molecules of different sizes: requirement for a specific high protein/RNA mass ratio. *J. Virol.* 86:3318–3326.
31. Goelet, P., G. P. Lomonossoff, …, J. Karn. 1982. Nucleotide sequence of tobacco mosaic virus RNA. *Proc. Natl. Acad. Sci. USA.* 79:5818–5822.

32. Strauss, E. G., C. M. Rice, and J. H. Strauss. 1984. Complete nucleotide sequence of the genomic RNA of Sindbis virus. *Virology.* 133:92–110.

33. Hyeon, C., R. I. Dima, and D. Thirumalai. 2006. Size, shape, and flexibility of RNA structures. *J. Chem. Phys.* 125, 194905.

34. Yoshikawa, K., M. Takahashi, …, A. R. Khokhlov. 1996. Large discrete transition in a single DNA molecule appears continuous in the ensemble. *Phys. Rev. Lett.* 76:3029–3031.

35. Birshtein, T. M., and V. A. Pryamitsyn. 1991. Coil-globule type transitions in polymers. 2. Theory of coil-globule transition in linear macromolecules. *Macromolecules.* 24:1554–1560.

36. Jacobson, D. R., D. B. McIntosh, and O. A. Saleh. 2013. The Snakelike Chain Character of Unstructured RNA. *Biophys. J.* 105:2569–2576, and references cited therein.

37. Grosberg, A., A. Gutin, and E. Shakhnovich. 1995. Conformational Entropy of a Branched Polymer. *Macromolecules.* 28:3718–3727.

38. Daimant, H., and D. Andelman. 2001. General Criterion for Controllable Conformational Transitions of Single and Double Stranded DNA. Preprint at arXiv. https://doi.org/10.48550/arXiv.cond-mat/0109253.

39. Katz, A. M., I. S. Tolokh, …, L. Pollack. 2017. Spermine Condenses DNA, but Not RNA Duplexes. *Biophys. J.* 112:22–30.

40. Kabir, A., and G. Suresh Kumar. 2014. Targeting Double-Stranded RNA with Spermine, 1-Naphthylacetyl Spermine and Spermidine: A Comparative Biophysical Investigation. *J. Phys. Chem. B.* 118:11050–11064.

41. Ruiz-Robles, J. F., A. M. Longoria-Hernández, …, J. Ruiz-Garcia. 2022. Spontaneous Condensation of RNA into Nanoring and Globular Structures. *ACS Omega.* 7:15404–15410.

42. Tellinghuisen, T. L., A. E. Hamburger, …, R. J. Kuhn. 1999. In Vitro Assembly of Alphavirus Cores by Using Nucleocapsid Protein Expressed in *Escherichia coli*. *J. Virol.* 73:5309–5319.

43. , See, for example Lomonossoff, G. P., and T. M. A. Wilson. 1985. Structure and In Vitro Assembly of Tobacco Mosaic Virus, Chapter 2 in. *Molecular Plant Virology.* 1:43–83.

Biophysical Journal, Volume *122*

Supplemental information

Long ssRNA undergoes continuous compaction in the presence of polyvalent cations

Ana Luisa Duran-Meza, Liya Oster, Richard Sportsman, Martin Phillips, Charles M. Knobler, and William M. Gelbart

SUPPORTING MATERIAL (SI)

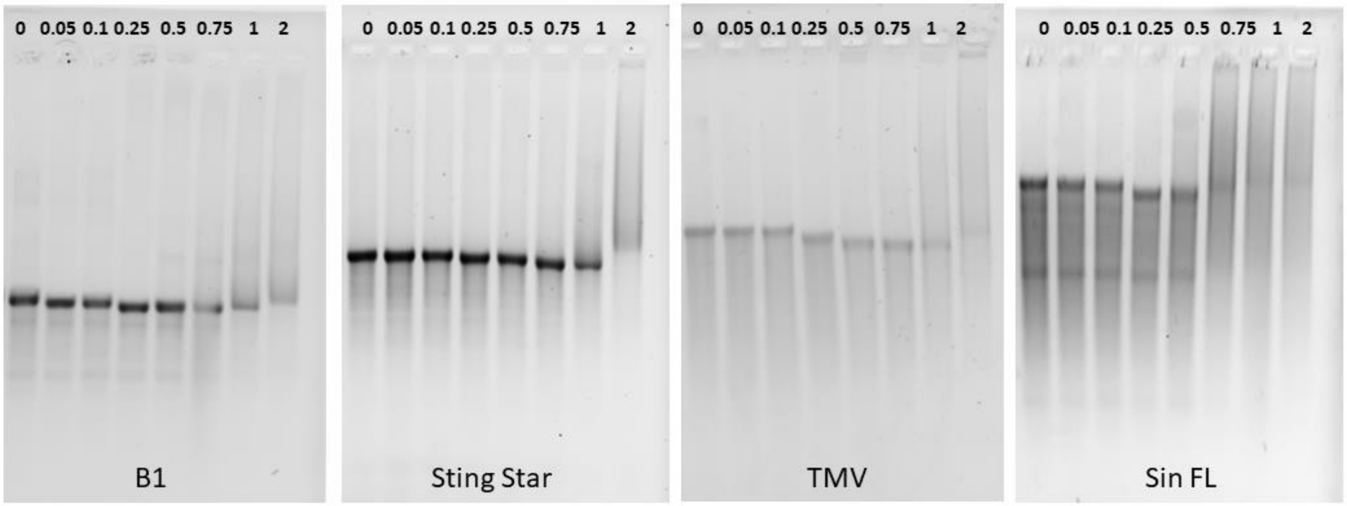

FIGURE S1 Eight-lane 0.8% agarose gels in TAE buffer, for (from left to right) B1 RNA (3234 nt), “Star” RNA (4628 nt), TMV RNA (6395 nt), and Sin FL RNA (11703 nt), for successively higher spermine:RNA charge ratios. Each gel contains 8 lanes labeled 0, 0.05, 0.1, 0.25, 0.5, 0.75, 1, and 2 for the spermine:RNA charge ratio. Note that in each case the molecules move faster with increasing charge ratio, because of their smaller size, until they begin to get stuck in the well at high enough charge ratio because of intermolecular aggregation.

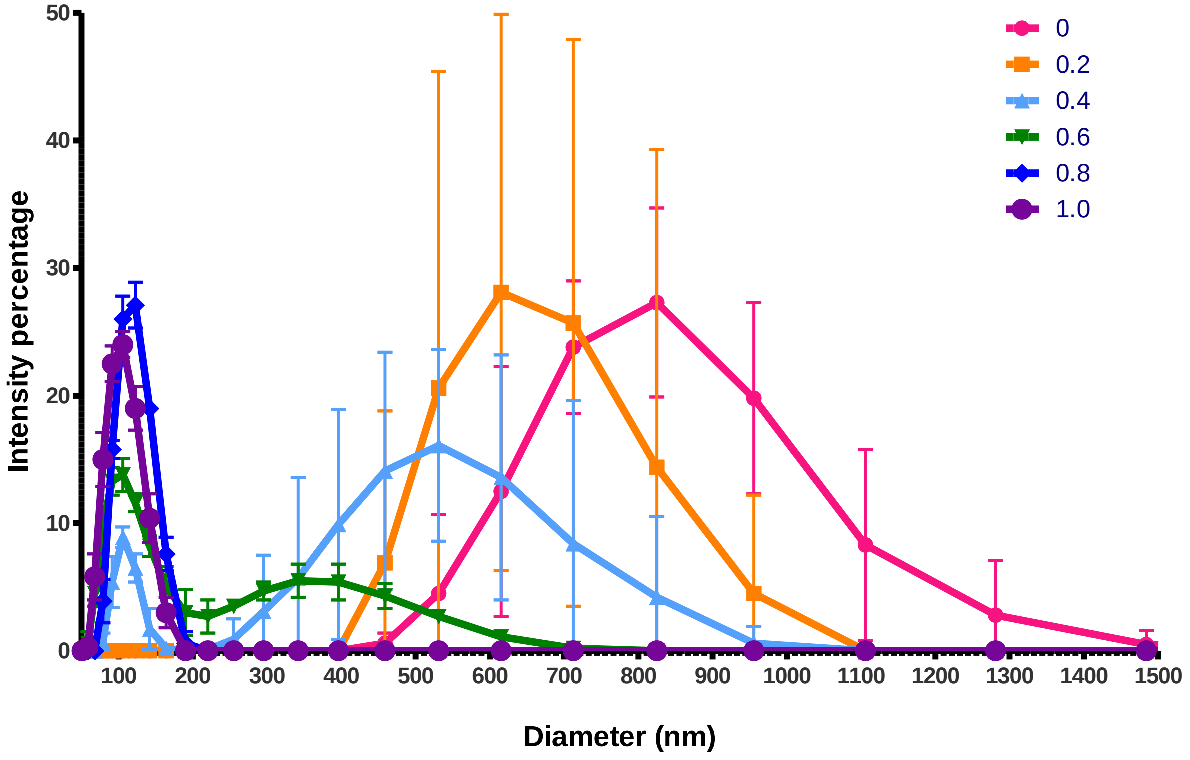

FIGURE S2 Size distributions measured by dynamic light scattering for Lambda DNA, for different spermine:DNA charge ratios, showing two-phase coexistence (bimodal distributions) for intermediate values (0.4:1 and 0.6:1), and unimodal distributions for higher (0.8:1 and 1:1) and lower (0:1 and 0.2:1) values.

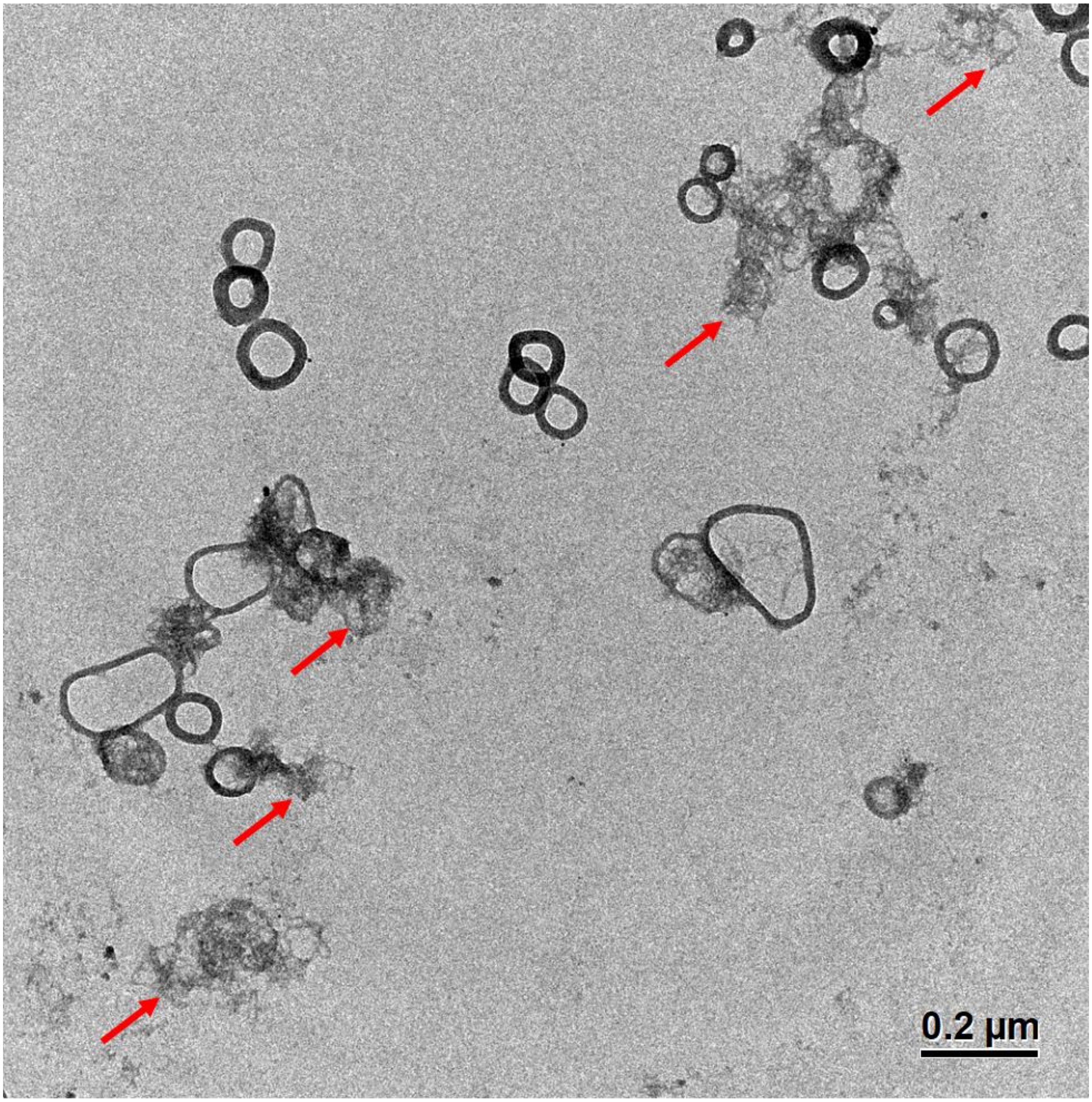

FIGURE S3 Electron micrograph of (48.5 kpb) Lambda DNA with a spermine:DNA charge ratio of 0.6:1, showing several instances, in the same field of view, of coexistence of uncondensed disordered DNA (see arrow) with strongly-condensed/toroidally-close-packed molecule**.** An aliquot of 6 μL of of DNA with spermine in TE buffer at a concentration of 0.2 mg/mL was deposited on glow-discharged carbon-coated copper (200-mesh) PELCO Pinpointer grids (Ted Pella, USA). After 1 m, the grids were blotted with Whatman filter paper, and then stained with 6 μL of 2 % uranyl acetate for 1 min followed by complete stain removal and storage in a desiccator overnight. Micrographs were acquired using a Tecnai G2 TF20 High-Resolution electron microscope (FEI, USA) with an accelerating voltage of 200 kV. Images were collected with a TIETZ F415MP 16-megapixel CCD camera (4000 by 4000 pixels, pixel size 15 μm).

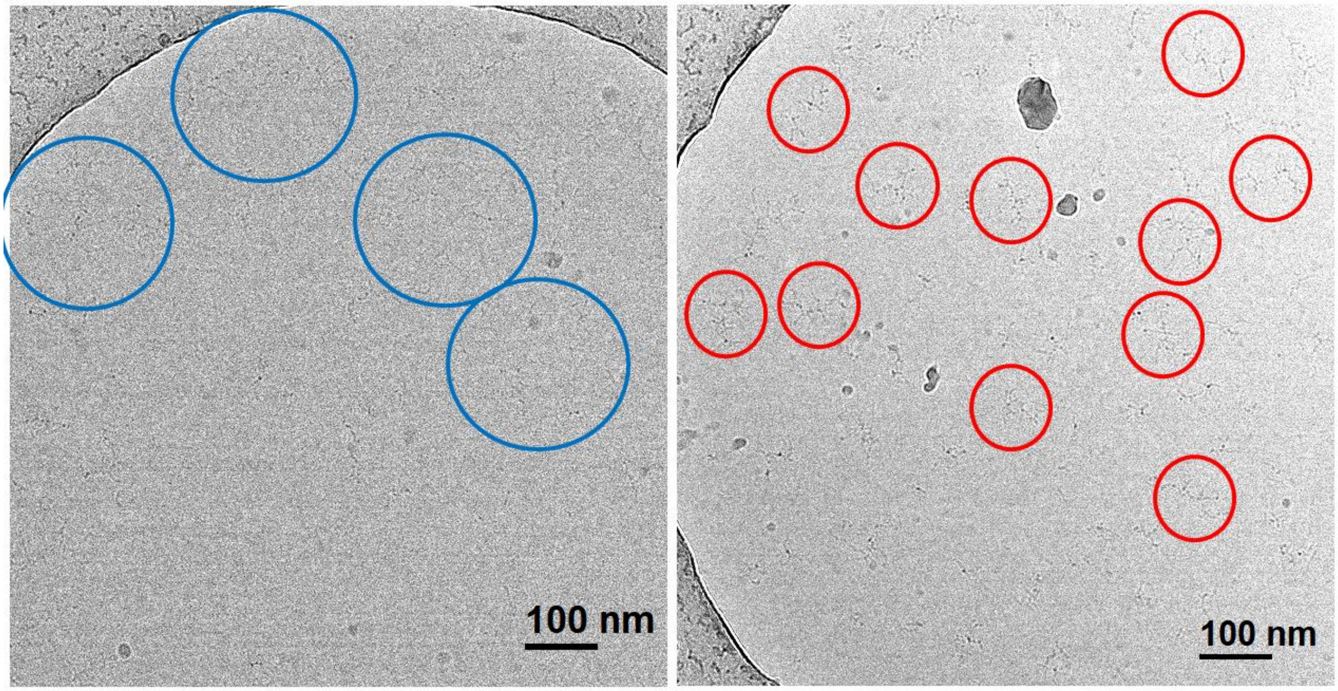

FIGURE S4 Electron micrographs of Sin FL ssRNA in the absence (left) and presence (right) of spermine (at a charge ratio of 1). Blue and red circles are guides-to-the-eye highlighting the differences in size of the molecule under these conditions of polyvalent cation. The molecules are barely visible because of their low density: no discontinuous condensation has occurred – rather, the only effect of the spermine is to progressively and weakly compactify the RNA. An aliquot of 3 μL of RNA with spermine in water at a concentration of 1 mg/mL was applied to a QUNTIFOIL holey carbon grid (2/1, 300 mesh), which was glow-discharged for 60 s with a PELCO easiGlow system.. The grid was blotted and plunged-frozen from 4°C under 100% humidity into liquid ethane with a Vitrobot IV. The grid was stored in liquid nitrogen until imaging. Cryo-Micrographs were acquired using a Tecnai G2 TF20 High-Resolution electron microscope (FEI, USA) with an accelerating voltage of 200 kV. Images were collected with a TIETZ F415MP 16-megapixel CCD camera (4000 by 4000 pixels, pixel size 15 μm).
